# Challenges and opportunities of ML and explainable AI in large-sample hydrology

**DOI:** 10.1098/rsta.2024.0287

**Published:** 2025-07-31

**Authors:** Louise Slater, Georgios Blougouras, Liangkun Deng, Qimin Deng, Emma Ford, Anne Hoek van Dijke, Feini Huang, Shijie Jiang, Yinxue Liu, Simon Moulds, Andrew Schepen, Jiabo Yin, Boen Zhang

**Affiliations:** ^1^ School of Geography and the Environment, University of Oxford, Oxford, UK; ^2^ Department of Biogeochemical Integration, Max Planck Institute for Biogeochemistry, Jena, Thüringen, Germany; ^3^ ELLIS Unit Jena, Jena, Thüringen, Germany; ^4^ State Key Laboratory of Water Resources Engineering and Management, Wuhan University, Wuhan, Hubei, People's Republic of China; ^5^ Department of Atmospheric Science, China University of Geosciences, Wuhan, Hubei, People's Republic of China; ^6^ Department of Atmospheric Physics, University of Oxford, Oxford, UK; ^7^ Louis Bolk Instituut, Bunnik, The Netherlands; ^8^ School of Atmospheric Sciences, Sun Yat-sen University - Zhuhai Campus, Zhuhai, Guangdong, People's Republic of China; ^9^ Geography and Environment, Loughborough University, Loughborough, UK; ^10^ School of GeoSciences, The University of Edinburgh, Edinburgh, UK; ^11^ Commonwealth Scientific and Industrial Research Organisation, Dutton Park, Queensland, Australia

**Keywords:** hydrology, machine learning, artificial intelligence, explainable AI, interpretable AI, XAI

## Abstract

Machine learning (ML) is a powerful tool for hydrological modelling, prediction, dataset creation and the generation of insights into hydrological processes. As such, ML has become integral to the field of large-sample hydrology, where hundreds to thousands of river catchments are included within a single ML model to capture diverse hydrological behaviours and improve model generalizability. This manuscript outlines recent advances in ML for large-sample hydrology. We review new tools in explainable AI (XAI) and interpretability approaches, as well as challenges in these areas. Key research avenues for large-sample hydrology include addressing variability in interpretations resulting from different ML models and XAI techniques, enhancing hydrological predictions in data-sparse and human-impacted regions, reducing the ‘cascade of uncertainty’ inherent in hydrological modelling, developing improved methods for multivariate prediction and identifying causal relationships.

This article is part of the Royal Society Science+ meeting issue ‘Hydrology in the 21st century: challenges in science, to policy and practice’.

## 1. Introduction: ML for large-sample hydrology

Artificial Intelligence (AI) and Machine Learning (ML) are powerful tools for generating skilful predictions relative to traditional hydrological models [1–5] and for uncovering insights into physical processes [6–8]. Many reviews have examined the application of ML in hydrology, comparing process-oriented and data-driven modelling approaches, and highlighting the spectrum of ML methods applied to areas like rainfall–runoff modelling, groundwater, water quality, floods and droughts [9–12]. Some of these reviews have focused on specific domains, including water resources management [13,14] and short- to long-term flood prediction [15], as well as techniques, including explainable AI (XAI) [16,17], ensemble learning methodologies [18] or meta-heuristic algorithms [19]. Additionally, these reviews have discussed some of the challenges of ML in hydrology, including the extrapolation problem, physical interpretability and limitations from small sample sizes or data scarcity (e.g. [9]).

Here, by contrast to previous reviews, we focus on recent developments at the intersection of large-sample (multi-catchment) hydrology and ML [2], with particular attention to emerging work in explainable and interpretable AI [8,20]. Large-sample hydrology leverages data from many river catchments to move beyond individual case studies and allow more robust conclusions about hydrological processes [21]. ML-based approaches in this subfield have advanced rapidly since the foundational work of Kratzert *et al.* in 2018−2019 [1,2,22]. Large-sample ML approaches train a single ML model on hydrological and meteorological time series from many catchments, integrating static attributes such as climate, topography, geology, and land cover. The inclusion of static attributes allows the large-sample model to learn how the rainfall–runoff relationship varies across different catchments [2,6]. A major advantage of the large-sample ML approach is that the broader training envelope reduces the likelihood of extrapolation relative to single-catchment analyses [23], thereby improving generalization [3–5] and enhancing the prediction of extremes [24]. Our focus is on new ML methods that are being developed to derive systematic process insights across many catchments. We do not seek to provide an exhaustive or technical catalogue of ML model architectures, tools or taxonomies. The field is developing so rapidly that such efforts would quickly become outdated. Instead, we take a narrative approach, outlining the key successes of ML and explainability within the field of large-sample hydrology. We illustrate this progress through selected examples from the literature.

Contrary to the widespread perception of ML models as ‘black boxes’, we show how advances in XAI, a set of advanced techniques to interpret machine learning models, are now providing greater insight into the relationships between different variables and model predictions, particularly when combined with a large-sample hydrological modelling approach. ML models learn patterns directly from the data, enabling them to reveal relationships that may not be captured by traditional models—provided the model is well designed and trained on suitable data [25]. We believe this field represents a significant opportunity for uncovering new hydrological insights in the future. However, large-sample ML models also face various challenges that may limit their adoption in the hydrological sciences. These challenges include data availability issues, such as the over-representation of data from Europe and North America, which can lead to biased models. Additionally, model training problems can occur such as shortcut learning [26], where models identify simpler patterns (shortcuts) in the training data that do not capture the true underlying relationships, leading to high performance on the original dataset but poor generalization to new data. Here we review the strengths of ML for large-sample hydrology (§2), approaches for generating novel insights through explainability and attribution of large-sample ML models (§3), challenges associated with ML and XAI in large-sample hydrology (§4) and areas for further research (§5).

## 2. ML for enhanced hydrological modelling, forecasting and variable estimation

Below, we highlight some of the main types of applications of ML within large-sample hydrology, including their strengths and limitations. Areas of focus include simulation and forecasting of hydrological variables (e.g. streamflow, soil moisture, snow water equivalent, evapotranspiration), generation of new datasets (e.g. from remote sensing, digital elevation models and *in situ* measurements), and novel applications of differentiable or physics-informed hydrological models.

### (a) ML for hydrological simulation

**Figure 1.**
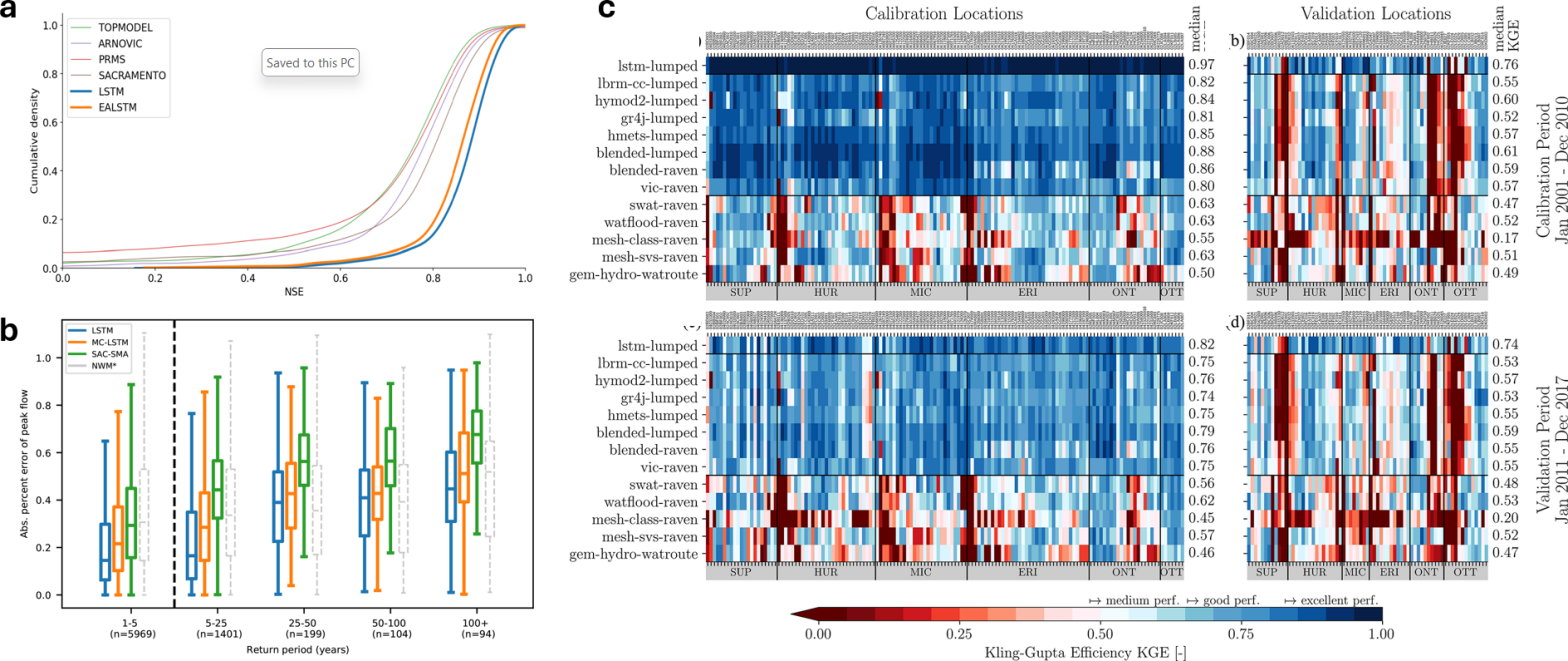
Benchmarking Long Short-Term Memory (LSTM) networks against traditional hydrological models. (a) LSTM and Entity Aware LSTM (EA-LSTM) are benchmarked against conceptual models (TOPMODEL, ARNOVIC, PRMS and Sacramento) in Great Britain; reproduced from Lees *et al.* [5]; (b) LSTM, Mass-Conserving LSTM (MC-LSTM), Sacramento Soil Moisture Accounting model (SAC-SMA) and the National Oceanic and Atmospheric Administration National Water Model (NWM), reproduced from Frame *et al.* [24]. (c) A suite of models including LSTM, basin-wise, subbbasin-based and gridded models with local to global calibration, reproduced from Mai *et al.* [4]. In panel (c), the performance is shown for each of the different models (*y-*axis) across the calibration and validation locations (*x-*axis).

It is now well-established that Long Short-Term Memory (LSTM) models trained on large samples of catchments typically outperform traditional hydrological models [2,4,5,24] (figure 1). LSTMs are a specialized form of recurrent neural network (RNN) in deep learning designed to handle sequential data effectively by capturing long-term dependencies, which traditional RNNs struggle with. They achieve this by mitigating the vanishing gradient problem, where gradients become too small during training, hindering the model’s ability to understand long-term dependencies in the data [27].

A robust model intercomparison in the Great Lakes region by Mai *et al.* found that an LSTM-lumped model significantly outperformed various types of basin-wise, subbasin-based and gridded models with local to global calibration, not only in calibration but also in every validation scenario [4]. While certain catchments remain difficult to model with LSTMs, such as those with spatially variable human impacts, Lees *et al.* showed that LSTMs exhibit considerable promise for learning intermediate stores such as soil moisture (see §3c), suggesting that during training the model implicitly learns certain hydrological processes that are explicitly defined in conceptual and physics-based models [5]. Addressing a common misconception about ML models, Frame *et al.* showed that LSTMs are consistently better at predicting extreme events when compared with conceptual, process-based and physics-informed ML methods, even when comparable extreme events are not included in the training dataset [24].

Compared with traditional hydrological models, a key strength of deep learning methods for continuous streamflow prediction is their ability to leverage multiple data streams to improve simulation performance. One notable example of this is the synergistic use of meteorological drivers from different forcing products. For instance, Kratzert *et al.* found that an LSTM could extract information from three different meteorological forcing products over the continental United States, dynamically weighting the information from each forcing product depending on location and flow conditions [28].

**Figure 2.**
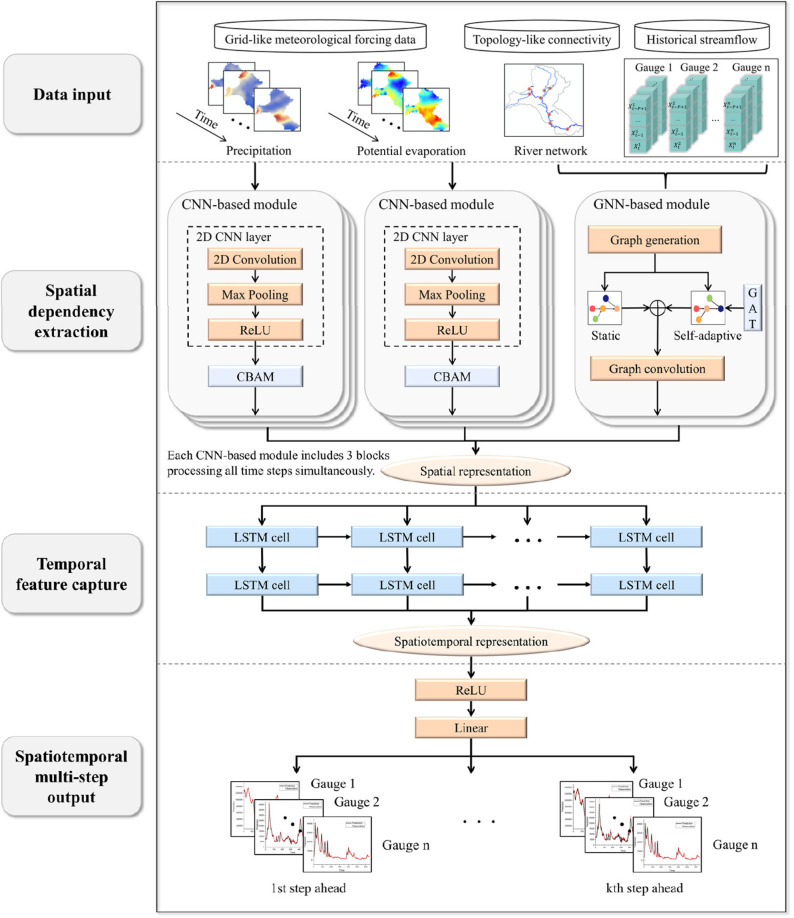
Spatio-temporal modelling. The CNN-based modules and GNN-based modules are employed to recognize spatial information from Euclidean gridded meteorological forcings and non-Euclidean irregular topological connectivity, respectively. The LSTM model is then employed to extract temporal dependencies. The multi-step and multi-gauge streamflow simulations are output through the subsequent ReLU function and linear layers. Figure reproduced from Deng *et al.* [29].

Furthermore, recent progress has revealed the ability of DL models to integrate different types of spatial data for hydrological modelling (figure 2). While existing DL models tended to use either Euclidean data, such as gridded meteorological forcing, or non-Euclidean data with irregular topological connectivity, such as information transfer between nodes along a river network, Deng *et al.* showed that the two types of information could be blended to systematically improve model performance [29]. They integrated Convolutional Neural Networks (CNN) for regular spatial data and Graph Neural Networks (GNN) for handling irregular data, along with spatial attention mechanisms to focus on important features, and an LSTM to capture temporal dependencies. With this model structure, the authors simulated streamflow at multiple lead times and observed performance improvements, particularly at extended lead times and at tributary gauges that were previously the most difficult to model.

### (b) ML for hydrological forecasting

The performance of ML models for streamflow prediction compared with traditional hydrological models means they are highly suitable for hydrological forecasting, where accuracy and reliability are primary concerns. In recent years, the Google Flood Forecasting team has built a suite of forecasting services based around LSTM neural networks to predict global flooding with up to 7 days lead time, demonstrating equal or better performance than the operational Global Flood Awareness System (GloFAS) [3]. In their approach, an encoder LSTM processes static geophysical data and 365 days of historical meteorological forcing data. The encoder’s states are passed to a decoder LSTM, which runs over 7 days of meteorological forecast data. The model outputs are time-step dependent parameters of an asymmetric Laplacian mixture distribution from which streamflow quantiles can be drawn. This approach enables streamflow forecasting directly from meteorological inputs in ungauged basins, with parameter prediction characterizing the uncertainty in the forecast.

A growing body of work has evaluated the advantages of ML models trained on dynamic forecasting model outputs to predict hydrological variability and extremes at subseasonal to seasonal and decadal lead times [30]. Here, a key research area has been to define the best approach to achieve good predictions given uncertain climate forecasts. Hauswirth *et al.* [31] compared multilinear regression, lasso regression, random forests and LSTM models trained on historic observations of seasonal discharge and surface water levels in single river catchments in the Netherlands. The predictions were generated using seasonal (re)forecast data. The authors found only minor differences between the various ML approaches and hypothesized this was due to the uncertainty in the forecast data outweighing the relative difference in performance of the ML algorithms. A key difficulty for hybrid forecasting, which combines machine learning models with dynamical forecasts [30], remains the limited skill of the dynamical forecasts months ahead. However, the limited benefit of the ML models may also arise from the lower performance of single-catchment machine learning approaches compared with large-sample (multi-catchment) approaches [23]. In the UK, a large-sample seasonal forecasting system was developed to predict monthly maximum daily streamflow up to four months ahead by training a quantile regression forest (QRF) model directly on dynamical seasonal forecasts of precipitation and temperature from a multi-model ensemble of C3S seasonal climate forecasts, allowing the ML model to implicitly perform bias-correction and downscaling. The multi-catchment ML model was marginally, but significantly, more skilful than the single-site approach [32]. Increasingly sophisticated ML approaches could potentially provide greater benefit over simpler ML models. However, in the absence of skilful climate forecasts, skilful long-range flood forecasts are unlikely to be achieved, no matter how sophisticated the machine learning model.

A challenge for the hydrological ML community is to develop skilful forecasting models that effectively convey forecast uncertainty, especially at longer lead times. While deterministic ML or DL models still remain the most commonly used approaches [33,34], there is a growing shift toward ensemble learning methodologies, including bagging, boosting, model averaging, and stacking [18]. Alongside these, probabilistic ML methods that directly estimate uncertainty [35,36] and neural network-specific uncertainty quantification techniques [37] are gaining traction (see §4c on uncertainty quantification). Large-sample hybrid forecasting approaches, which combine ML with the outputs of dynamical forecasting models, are starting to address uncertainty quantification through probabilistic methods [30,38], as exemplified by Google’s approach of distributional parameter prediction [39].

### (c) ML for generating hydrological datasets

ML is increasingly used for generating new datasets or predicting variables which are limited by record length or sparsely distributed in space. For instance, a ML-based reconstruction of global terrestrial water storage (GTWS-MLrec) was used to extend the record length of TWS data back to 1940 [40], allowing a better understanding of multidecadal dynamics of the terrestrial water budget than permitted by GRACE/GRACE-FO data alone. ML has also been used to develop static datasets of physical parameters that are important for hydrological modelling and analysis. For example, bankfull river discharge was predicted for millions of kilometres of streams and rivers globally using a random forest model trained on multiple static attributes [41]. Other variables include the prediction of global river width [42] or river flood depth and extent [43]. Such datasets are particularly relevant for hydrodynamic modelling, where they help capture flow and inundation dynamics, but they may also be interrogated to understand broader hydrological and morphological patterns, or used to parameterize or validate process-based models. ML is also widely used to predict discrete hydrological variables such as streamflow signatures [8,44] and the characteristics of extreme events. Random forests are particularly suited to these applications because of their relative resistance to overfitting and ability to describe nonlinear relationships between the features and the predicted variable through post hoc explainable AI tools (see §3) [45].

### (d) Differentiable, physics-informed ML models for hydrology

Different approaches exist to combine ML with physical information, by adding synthetic input data, imposing constraints on ML models and blending ML models with process-based models. First, in cases where ML models perform poorly in predicting the most extreme events such as floods and droughts, synthetic atmospheric data can be included to help the models learn the patterns associated with these extremes. Synthetic samples have been shown to effectively improve the ability of ML models to simulate extreme events [46]. Second, ML models can ensure adherence to physical conservation principles, such as water and energy balance, by incorporating corresponding constraints into the loss functions as penalties. An example of lake temperature simulation after introducing energy conservation into the loss function proved to be physically consistent in the net thermodynamic fluxes into and out of the lake [47].

In addition, constraints can also be imposed on the structure of ML models. Zhao *et al.* implemented physical constraints (a modified Penman–Monteith equation) into the structure of an artificial neural network model to predict latent heat flux [48]. They showed the benefit of this hybrid physics-informed neural network was that it could conserve the surface energy budget, respect the physics of evaporation and better generalize during extremes. Recent work has also explicitly tested the value of using strictly enforced mass conservation constraints to model the rainfall–runoff process. Frame *et al.* [49] demonstrated that using such constraints could actually harm hydrological modelling due to errors in the precipitation and streamflow data. Deep learning models learn to account for data biases, and the ‘closure’ effect contributes only minimally to the difference in predictive skill between data-driven and conceptual models. A different approach, also referred to as physics-informed but focusing on incorporating causality (rather than imposing physical equations in the structure of ML models), is the approach taken by Adombi *et al.* [50]. The authors imposed causal relationship constraints (CRCs) on the layers of the model to enforce learning of causal relationships. This allowed them to explain future changes in groundwater levels based on the changes in vertical inflow and potential evapotranspiration under different climate scenarios.

Third, hybrid methods that blend DL with conceptual hydrological models are increasingly popular to preserve specific process-based components such as the relationship between soil moisture and runoff generation. For instance, a conceptual hydrological model (GR4J) was blended with deep learning models to preserve the GR4J production storage processes and then route runoff based on net rainfall and runoff from the production storage [51]. By incorporating DL, other time series such as temperature and antecedent streamflow could be included to improve predictive performance. The hybrid approach performed better than either the conceptual model or DL alone. Temporal dynamic models, often employed in the field of hydrology, can be inserted into an AI system as special recurrent neural layers to improve the representation of the physical system. This kind of physics-informed paradigm improves simulation accuracy, transferability and unobserved process inference [52].

**Figure 3.**
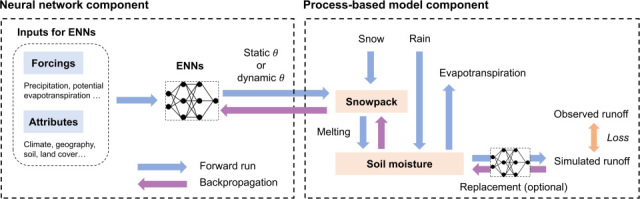
Structure of a differentiable hydrological model. A neural network receives dynamic inputs from meteorological forcings and static attributes across a large number of basins to provide regionalized parameterization for the process-based model. The hydrological parameters can be either static or dynamic. Modified from Shen *et al.* [54].

As another genre of physical-informed hybrid model, differentiable models are generally composed of a process-based model (or a DL surrogate), which provides physical constraints, and embedded neural networks which can learn the model parameters or replace any internal modules of the physical model. For instance, a differentiable model based on the HBV conceptual hydrological model was used to develop a regionalized model parameterization [53], with the original soil moisture–runoff relationship replaced with an inserted neural network to learn the relationship between soil moisture, precipitation and runoff (figure 3). These models are called ‘differentiable’ because the gradients of the physical model outputs are tracked with respect to the inputs using differentiable programming. This blending approach allows them to be framed either as ML models constrained by structural priors, or process-based models enhanced by learnable units [54]. While their temporal performance approaches that of LSTMs, one of their strengths is that they can simultaneously respect mass conservation and output a suite of interpretable uncalibrated internal physical fluxes and state variables, such as evapotranspiration and soil moisture [53,54]. In addition, with the assistance of learnable units, differentiable models exhibit competitive or even better spatial generalization when predicting in ungauged basins and ungauged regions [55]. These advantages are particularly relevant in predicting high flow trends in ungauged locations, where the model’s treatment of hydrological extremes is grounded in physics. Explicit encoding of physical models in neural networks also allows data from multiple processes or subsystems to be assimilated in a physically consistent manner. For instance, integrating hydrological and dynamic vegetation modelling into differentiable models can significantly improve the spatio-temporal representation of evapotranspiration in catchments by accounting for the two-way interactions between vegetation and hydrology [56]. Likewise, the explicit inclusion of river routing processes in the differentiable model allows for the effective use of streamflow observations from different gauges along a river network, thereby enabling hydrologically meaningful distributed modelling with ML [57,58].

## 3. Generating novel hydrological insights through model explainability and interpretability

One of the key challenges for hydrologists is using ML to derive new knowledge and findings. Hydrology generally seeks to quantify relationships within the data, identify influential variables and understand the nature of their influence (linear, nonlinear or conditional). XAI plays a key role in equipping hydrologists with tools to measure these relationships. Specifically, XAI methods allow for the analysis of the relationships learned by complex ML models, evaluating model components or sensitivities, and thereby helping to generate new hypotheses about the underlying mechanisms [25]. Multiple approaches exist for evaluating the relative importance or contribution of feature variables within an ML model, but the explanation obtained can vary depending on the technique used. It is thus essential to understand exactly how each technique works. Some methods are specific to certain models (i.e. intrinsic) while others can be applied to different ML model types (i.e. post hoc). We focus principally on post hoc approaches which can be applied to different types of ML models. Many XAI methods rely on a perturbation-based approach, whether implicitly through post hoc interpretability methods such as relative importance, Partial Dependence Plots (PDPs), Individual Conditional Expectations (ICEs), Accumulated Local Effects (ALEs) or SHAP, discussed in §3a, or more explicitly through hypothesis testing and sensitivity testing, discussed in §3d.

### (a) Model-agnostic post hoc explainability methods

**Figure 4.**
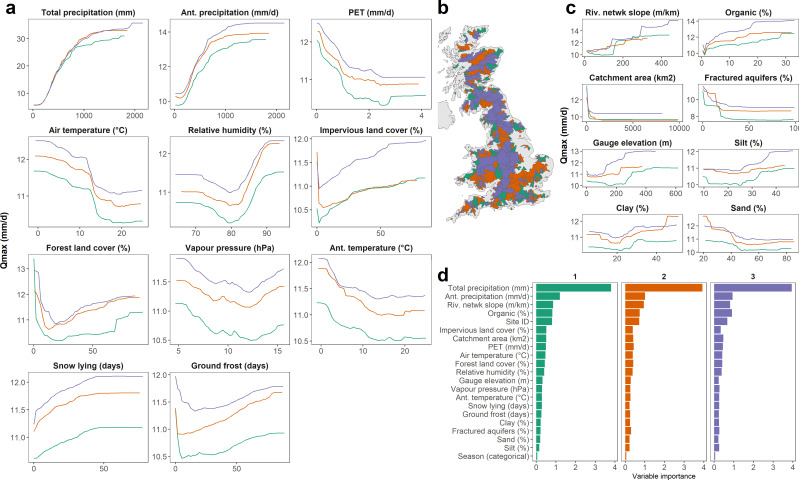
Partial dependence plots for a quantile regression forest (QRF) large-sample model with 1268 gauges across Great Britain. Catchments are randomly split into three groups (3 colours) to assess consistency of partial dependence plots (panels a–c) and relative importance (panel d) across the three separate models. Features include time-series variables (a) and static catchment attributes (c). Reproduced from Slater *et al.* [8].

**Figure 5.**
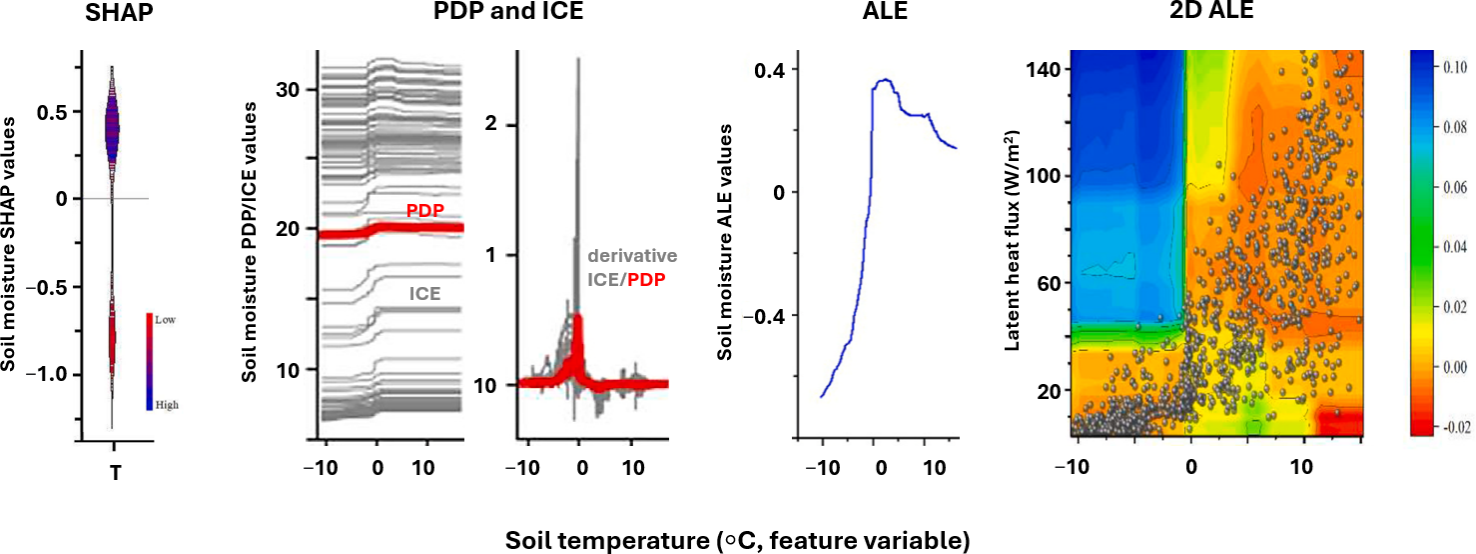
Four XAI metrics showing the effect of soil temperature (°C, feature variable) on the predicted soil moisture (target variable). The four sets of plots indicate (1) distribution of SHAP values, with red colours indicating low feature values (low soil temperature) and blue colours high values (high soil temperature) (2); Partial Dependence Plot (PDP, red line) and Individual Conditional Expectations (ICE, grey lines) on same plot, along with derivative PDP and ICE (3); Accumulated Local Effects (ALE); and (4) two-dimensional ALE plot, with soil temperature on the *x*-axis, latent heat flux on the *y*-axis and soil moisture as colour bar. Modified from Huang *et al.* [7].

In this section, we provide an overview of some key, model-agnostic XAI techniques, and we discuss issues and challenges which may arise from their use. The different methods described below all provide a form of relative feature (i.e. predictor) importance. Some techniques describe the magnitude of that effect (e.g. relative importance; figure 4), while others describe the shape of the association between a predictor and the target variable—i.e. PDPs, ICEs and ALEs (figure 5). It remains important to remember that XAI methods do not explain the physical reality but the ML model’s representation of the physical reality.

#### (i) Relative importance

Relative importance describes the contribution or influence of each feature in the model’s predictions, helping to understand which of the features are more influential in predicting the target variable (e.g. figure 4 from [8]). For instance, in a large-sample analysis of river mobility in 48 rivers worldwide, Leenman *et al.* employed relative importance to show that discharge variability was the primary predictor of river mobility relative to mean water discharge, sediment concentration and channel-bed slope [59]. Random forests have a built-in approach to compute relative importance [60], which is conceptually similar to R2 decomposition in linear regression [61]. Permutation importance is the most simple to implement and understand; it provides an intuitive measure of feature importance by observing the effect of randomly shuffling the features. This also provides insights for feature engineering decisions, as features with low importance can be removed in cases where the model needs to be simplified. However, permutation importance can be computationally expensive with large datasets, can provide misleading importance in the presence of correlated features, and can be unstable if the model is not robust. By contrast, feature importance from tree-based models (such as Gini importance) is derived directly from the model training process, making it more computationally efficient. However, it can give more importance to predictors with more categories, and can also be misleading in the presence of correlated features. Another point worth noting is that relative importance approaches provide a global summary of feature importance (i.e. in the full model), ignoring potentially important local variability. Moreover, feature importance techniques only provide information on the importance of a given variable and not the direction of a relationship between a feature and the outcome. It is thus valuable to combine relative importance with other interpretation methods for greater insight.

#### (ii) Partial dependence, ICE and ALE

Three techniques are listed here which provide information on the nature of the relationship between a feature variable and the predicted outcome. Outside of these ML techniques, such relationships can also be described with statistical summary tools such as elasticity curves, which depict, for instance, streamflow sensitivity to precipitation across the entire flow distribution [62].

Partial Dependence Plots (PDPs) provide an intuitive visualization of the relationship between an input feature and the target prediction within an ML model. They show the marginal effect of the feature on the predicted outcome of the model (i.e. how a change in the feature affects the prediction, while averaging out the effect of all other features). For instance, in figure 4*a*, increases in precipitation are associated with increasing flood magnitude [8]. PDPs can also show the effect of pairs of features on the target variable while keeping all other features fixed. One of the key limitations of PDPs is that they assume each feature of interest is independent of the other features, an assumption which may lead to potentially misleading interpretations when features are correlated. It is therefore worth assessing how the PDPs change when a correlated variable is removed from the model. Additionally, they only show the average effect of the feature in the model, rather than the full range.

Individual Conditional Expectation plots (ICEs), which visualize how changes in a particular feature influence the model’s prediction for individual instances, can thus provide greater insight than the PDP [63]. ICEs help detect heterogeneity in the model’s predictions, help identify outliers and extreme cases, reveal interactions between features, and enhance overall model interpretability. In figure 5, the grey lines show the ICEs of individual instances within the data while the red line shows the PDP, which displays the average effect across all features.

Accumulated Local Effects (ALE) are also used to assess a feature’s marginal effect on the predicted outcome. ALEs are considered more robust than PDP because they do not assume feature independence. Figure 5 shows that temperature is one of the key features affecting the predicted soil moisture using various interpretability techniques. When the temperature reaches 0°C, the thawing rate increases and the snowmelt leaches into the soil, significantly increasing the soil moisture. The derivative ICE of temperature shows a surge around 0°C in accordance with the ICE and ALE. The two-dimensional ALE of temperature and latent heat flux shows the distribution of instances as dots. As the temperature increases above 0°C (*x*-axis), the soil moisture decreases (colour bar), while latent heat flux rises. This can be explained by the joint effect of latent heat flux and temperature, both of which have a negative impact on soil moisture above freezing [7].

#### (iii) SHAP: the additive explanations approach

SHapley Additive exPlanations (SHAP) is an additive variable attribution method used to make models interpretable (see figure 5). By contrast with the methods described above, SHAP provides additive explanations, meaning the prediction can be expressed as the sum of the feature contributions. For instance, if the prediction is streamflow, the SHAP value for each predictor represents the contribution of that predictor to the deviation of the streamflow prediction from the average streamflow. In other words, the sum of SHAP values equals the model’s output difference from the baseline prediction.

SHAP is considered more robust than other relative importance measures due to its fair handling of feature contributions. Derived from game theory, SHAP values are calculated based on each feature’s contribution to the prediction across all possible combinations of feature subsets, ensuring consistent and fair explanations. Correlated features are handled more robustly by distributing contributions among features based on their marginal contributions. SHAP allows for the interpretation of both the direction and the magnitude of the impact of each feature on the target variable prediction, and provides insights at the global level (overall model behaviour; SHAP summary plots) and the local level (individual predictions; SHAP dependence plots).

For example, De Meester & Willems [64] used SHAP summary plots to assess four drought metrics, including drought intensity, drought severity, number of dry days and summer volume, to reveal that the proportion of irrigation in the catchment was one of the leading contributing factors of drought intensity and summer flow volumes in Flanders, Belgium. It is also possible to explicitly compute the interaction effects between features, to assess their combined effects after accounting for individual contributions, through SHAP interaction values [65]. These values are represented as a matrix for each sample with dimensions M×M, where M is the number of features. Jiang *et al.* [20], for instance, used the variability of SHAP interaction values across different flood sizes to assess the complexity of flood generation processes in individual catchments, noting that this complexity is likely to undermine the reliability of traditional flood frequency analyses.

### (b) Gradient-based interpretability methods

Various other methods besides the post hoc model-agnostic XAI methods described above can be used to interpret ML models. Gradient-based methods quantify the sensitivity of model predictions to small changes to the input features. They embed core explainability principles of sensitivity (features that have the most influence on model predictions are assigned greater importance) and completeness (the total contribution of all features explains the model’s output), ensuring they generate accurate and meaningful explanations [66]. They are popular methods for interpreting neural networks because the gradients can be retrieved through a backward pass after training.

Integrated and expected gradients are two approaches used for interpreting DL models. In integrated gradients (IG), the prediction of the DL model is attributed to its input features by computing the integral of gradients of the model’s prediction with respect to the input along a straight path from a baseline (often zero) to the input [67]. This results in an attribution score for each input feature. Expected gradients (EG) are a variant of IG, where the attribution is averaged over multiple samples across a distribution of inputs, reducing the sensitivity to any single baseline in complex models or when dealing with noisy or diverse data distributions to provide a more robust attribution [68].

**Figure 6.**
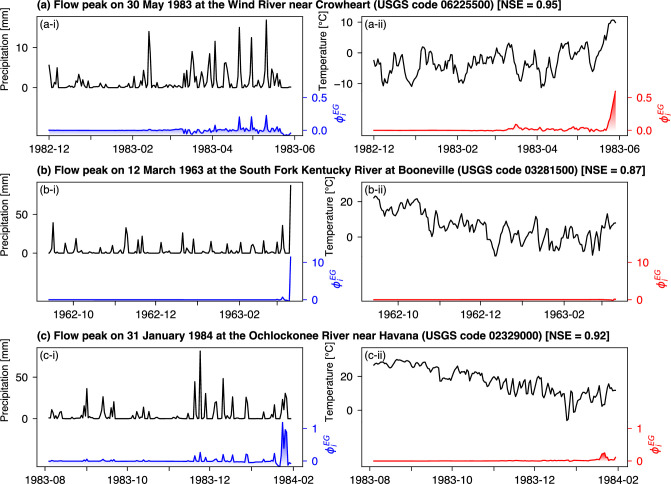
Observations (black lines) and feature importance scores (i.e. expected gradients; colour lines) of precipitation and temperature for three representative discharge peaks from three U.S. catchments. Each panel indicates the average NSE value of the target catchment during the test period in square brackets. Reproduced from Jiang *et al.* [69].

Gradient-based methods have been used, for instance, to interpret LSTM models that predict streamflow based on meteorological drivers [2,28], revealing temporal feature importance scores for precipitation and temperature in relation to annual maximum discharges [69,70]. Figure 6 exemplifies three distinct temporal patterns of these feature importance scores (coloured lines) for flood events from different U.S. catchments. In the first pattern (figure 6*a*), precipitation (blue line) slightly influences the peak discharge over an extended period, while temperature (red line) is more influential near the peak. The second pattern (figure 6*b*) shows minimal temperature impact and significant precipitation influence only near the peak. In the third pattern (figure 6*c*), precipitation has a sustained impact before the peak, suggesting historical precipitation may contribute significantly to peak flow. These patterns are consistent with three known flooding mechanisms—snowmelt, recent precipitation and antecedent precipitation (also known as excess soil moisture). This approach innovatively identifies flood mechanisms that emerge from complex interactions among flood drivers, without the (often subjective) classification criteria previously required, and supports further analysis of how climate and land surface shape these mechanisms and how they may change under global warming.

There are several other types of gradient-based methods that have been applied to CNN-based models. These include saliency maps [71], guided backpropagation [72], gradient × input [73], smooth gradients [74] and layer-wise relevance propagation (LRP) [75]. Given gridded input features, these methods can relate the model output back to each pixel of an input feature. This generates a saliency map or heatmap that allows for the identification of the input regions that are most crucial for the model’s output, which is particularly well-suited for physical inference in the geosciences.

### (c) Using latent variables to assess learned processes

**Figure 7.**
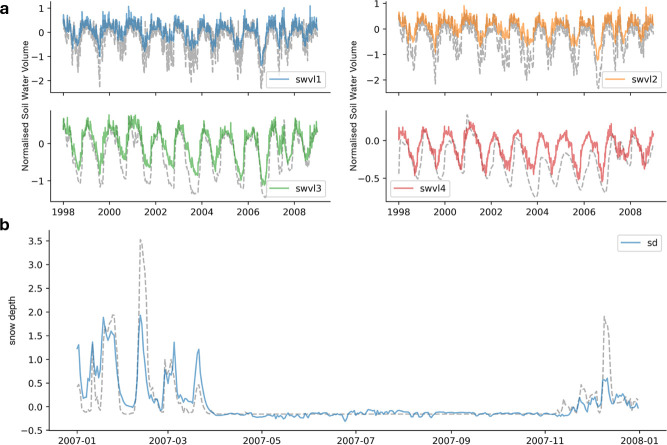
Using probes to explore hydrological variables learned by an LSTM model during model training. Panel (*a*) shows probe prediction time series as coloured lines alongside the target variables (grey dotted lines) for the Read Brook catchment at Hookagate, for four soil moisture levels. The probe correctly captures the temporal dynamics of the soil moisture signals, despite some systematic bias. Zero defines the mean soil moisture across Great Britain. Panel (*b*) shows probe predictions for the snow depth target variable at one site located in the Cairngorm Mountains. Reproduced from Lees *et al.* [6].

Latent variables offer an alternative approach to understand what a DL model has learned during training. They represent hidden factors or underlying structures in the data that the model has inferred but not observed directly. By analysing these latent variables it is possible to gain insights into the model’s internal representations and how it processes and interprets the data. For instance, Lees *et al.* designed an experiment to assess whether the learned relationships of an LSTM for streamflow simulation could be related to specific hydrological processes [6]. By extracting the tensors (i.e. the learned relationship), they assessed the hypothesis that the LSTM had learned a real world process. The cell-state vector, which represents the memory of the LSTM, was mapped to soil moisture and snow. The high correlation between the probe outputs and soil moisture/snow showed that the LSTM had learned the governing hydrological processes (see figure 7). Similar results were found by Jiang *et al.*, who trained a physics-informed ML model on streamflow observations but could still accurately infer catchment-wide snow dynamics through one of the recurrent NN’s intermediate cell-states [52].

### (d) Perturbation-based methods: hypothesis testing and sensitivity testing

**Figure 8.**
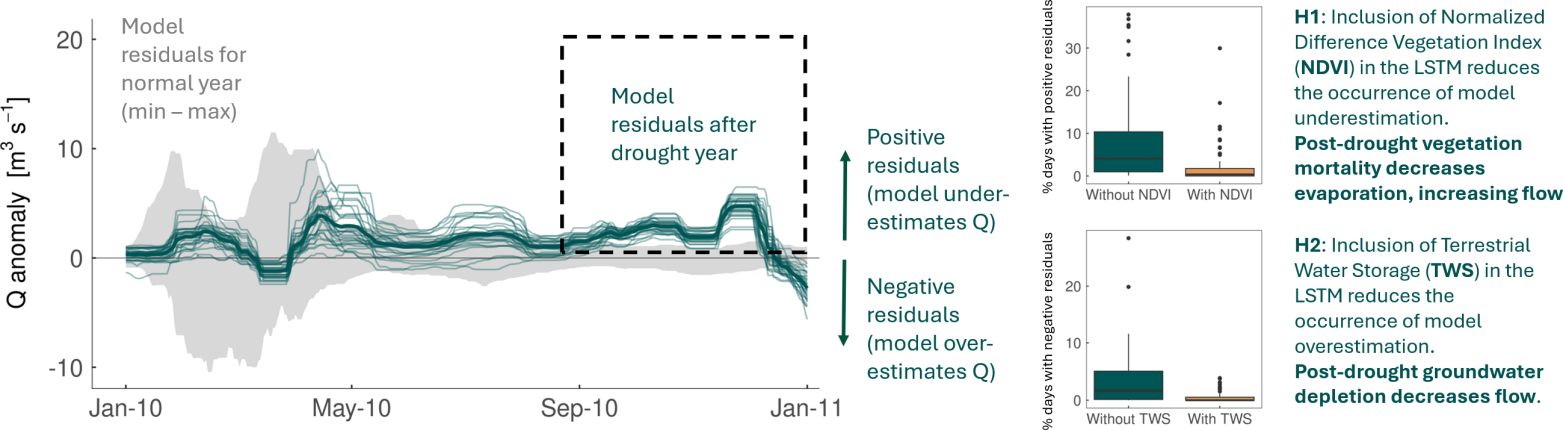
Hypothesis testing the causal mechanisms of post-drought streamflow legacy effects using a DL model. Grey shading shows model residuals in normal years; green lines indicate model residuals in drought years. Positive model residuals here (green lines enclosed in dashed black rectangle outline) show cases of the model underestimating streamflow in post-drought years. Top boxplots show that the inclusion of NDVI in post-drought years decreases the magnitude of residuals, reflecting the reduction in evaporation caused by vegetation mortality. Bottom boxplots show that the inclusion of TWS in post-drought years also decreases the magnitude of residuals, reflecting the decrease in streamflow from groundwater depletion. Example modified from Hoek van Dijke *et al.* [76].

One intuitive approach for analysing ML models is through model perturbation, where input features are systematically removed or replaced with permuted or randomly subsampled values to see how the outcome changes. Such approaches have been used to test the role of different driving mechanisms. For example, Hoek van Dijke *et al.* [76] used a DL-based approach to derive insights about driving mechanisms via hypothesis testing. They evaluated whether streamflow typically increased or decreased across different catchments of the globe in the years following a major drought, i.e. producing a ‘drought legacy effect’ on streamflow. They formulated two hypotheses: (i) that streamflow would increase following a drought in cases where drought-induced vegetation mortality decreased catchment evaporation, and (ii) that streamflow would decrease following a drought in cases where the groundwater was depleted. To assess these hypotheses, they first trained an LSTM model to predict streamflow from multiple variables, omitting the drought legacy years from the training data (following [77]). They then calculated the drought legacy effect based on the positive/negative model error in the drought legacy year (see figure 8). They found that in catchments with widespread vegetation mortality following a drought the inclusion of Normalized Difference Vegetation Index (NDVI) data in the LSTM model decreased the occurrence of model underestimation compared with the model without NDVI, suggesting that the observed increases in streamflow were caused by reductions in evaporation from vegetation mortality. Likewise, in catchments with depleted groundwater resources, they found that the inclusion of terrestrial water storage (TWS) in the model decreased the occurrence of model overestimation compared with the model without TWS, suggesting that the decreases in streamflow following droughts were largely due to groundwater depletion [76].

Aside from hypothesis testing, another approach to understand the model predictions is model sensitivity testing. This is a form of perturbation analysis. For instance, Slater *et al.* developed a quantile regression forest model across 1268 UK NRFA catchments and used this model to quantify the sensitivity of flood magnitude to a 10% increase in precipitation, a 1°C rise in air temperature, or a 10 percentage point increase in urban or forest land cover within a catchment [8]. The results of the sensitivity testing showed which catchments were more sensitive to changes in climate and land cover, revealing that increases in precipitation and urbanization tended to amplify flood magnitudes more in catchments that had high baseflow contribution, while rising air temperature and afforestation decreased flood magnitudes more in the catchments with low baseflow index.

## 4. Challenges associated with ML and XAI in hydrology

### (a) Causality in ML models

Most supervised ML models are built to leverage patterns in the data rather than to specifically identify causal relationships. In general, predictive models based on supervised ML focus on estimating the observational probability by predicting the likely values of Y when X takes on a certain value. By contrast, causal tasks aim to determine the interventional probabilities, to assess the impact of changes or interventions in X (e.g. setting X to a specific value) on Y [78]. A critical requirement for a predictive model to estimate causal effects is that confounding variables—affecting both the predictor and the target—are properly accounted for. If these confounders are not controlled, it becomes difficult to determine whether a given variable is a cause, an effect or unrelated to the target variable [79].

Several promising methods are emerging to attempt to assess causality with ML models, but they are not yet widely applied in hydrology. Causal ML methods which formalize the data-generation process through structural causal models (SCMs) have surged in recent years in other disciplines [80]. The causal random forests approach [81] can be implemented to estimate quantile treatment effects nonparametrically based on the generalized random forests method, along with a measure of variable importance. Counterfactual approaches, i.e. hypothetical retrospective interventions to explain an outcome, are also increasingly popular in the causal ML literature. Some causal ML approaches applied to soil moisture–precipitation coupling include a causal inference approach by Li *et al.*, who combined Granger causality analysis and ML [82] and a new methodology proposed by Tesch *et al.*, who trained a DL model to reflect causality using prior knowledge on additional variables that might affect the causal relationship [83]. The authors also suggested two additional approaches to assess whether the detected causality was more than a spurious outcome.

Although causal explanations remain the holy grail, there is still no existing approach for uncovering process mechanisms with certitude. Causal inference methods are not specific to ML and have various challenges such as contemporaneous causation, hidden confounding and nonlinearity—especially in the context of time series modelling [84]. Challenges in causal ML include the loss of generalization performance when the data distribution shifts [80]. In hydrology, this may occur when the hydrological models are trained on observations, but the forecasts use predictor variables with a different distribution, such as dynamical forecasts. Another key challenge when using XAI to uncover causality lies in the fact that ML models capture correlations and patterns in the data (such as nonlinear relationships between the target variable and predictors), but these relationships do not necessarily represent true causal relationships. Thus, while XAI tools can help interpret some of these patterns, providing insights into model behaviour and decision-making processes, they often struggle to distinguish between correlation and causality. More rigorous causal inference techniques are required to reliably separate spurious correlations from genuine causal effects.

### (b) Prediction in ungauged catchments/regions

Prediction in ungauged basins remains a longstanding problem in hydrology. Early applications of ML to address this challenge involved techniques for parameter regionalization [85], but the use of LSTMs delivered a major step forward. For example, Kratzert *et al.* [22] showed that an LSTM outperformed a conceptual model calibrated to a specific catchment, even when that catchment was left out of the data used to train the LSTM. In the Great Lakes Intercomparison Project [4], LSTM models systematically outperformed conceptual and physics-based models on the same test datasets (figure 1). Apart from LSTM models, random forest and differentiable models have also shown good performance and stability in data-scarce regions [41,55].

The performance of ML models in ungauged basins varies depending on the composition of the training dataset in relation to the ungauged basins. For instance, Fang *et al.* [86] showed that the highest predictive performance was achieved when the training dataset was representative and heterogeneous—a notion they termed ‘data synergy’. Kratzert *et al.* [23] observe that, in order to train as accurate as possible rainfall–runoff LSTM models, hydrologically diverse data from at least hundreds of basins should be employed, even if the geographical area of interest is limited. This contrasts with conventional hydrological modelling approaches where models are tailored to specific regions or regimes of interest. The implication is that hydrological predictions tend to improve in ungauged locations as the diversity of training data is increased.

Many large-sample (multi-catchment) machine learning applications in hydrology rely on pre-compiled datasets with static attributes, such as the ‘CAMELS’ datasets [87]. The performance of AI models in ungauged locations is typically assessed by evaluating the model on a subset of test basins that were left out during training. However, there are significant gaps in global coverage of these datasets, especially in Africa and Asia. Numerous efforts are underway to address this data gap, including the deployment of low-cost sensor networks and the use of satellite altimetry aboard missions such as SWOT [88]. Nevertheless, the question of how best to combine these novel data sources with AI to improve the accuracy and coverage of streamflow estimates worldwide remains an open question.

### (c) Uncertainty quantification in hydrological ML models

Probabilistic methods are gaining traction in machine learning for hydrology [35,36], with entire families of ML regression algorithms designed to provide probabilistic predictions, as summarized in figure 9 from [35]. These families include: (i) quantile regression algorithms that can support conditional quantile estimation (including quantile regression LSTMs, XGBoost and more). Most of these algorithms are based on the idea of using a ‘pinball loss’ function, rather than typical (mean) regression algorithms. (ii) Expectile regression algorithms are similar to quantile regression algorithms, except they focus on conditional expectiles rather than quantiles, and are slowly emerging in hydrology. (iii) Distributional regression algorithms (i.e. parametric algorithms) are expected to exhibit better skill than the first two types when sufficient information about the required predictive probability distribution is available. Papacharalampous and Tyralis highlight that the relative performance of these algorithms depends largely on the challenge that needs to be solved, as well as their degree of interpretability and flexibility.

**Figure 9.**
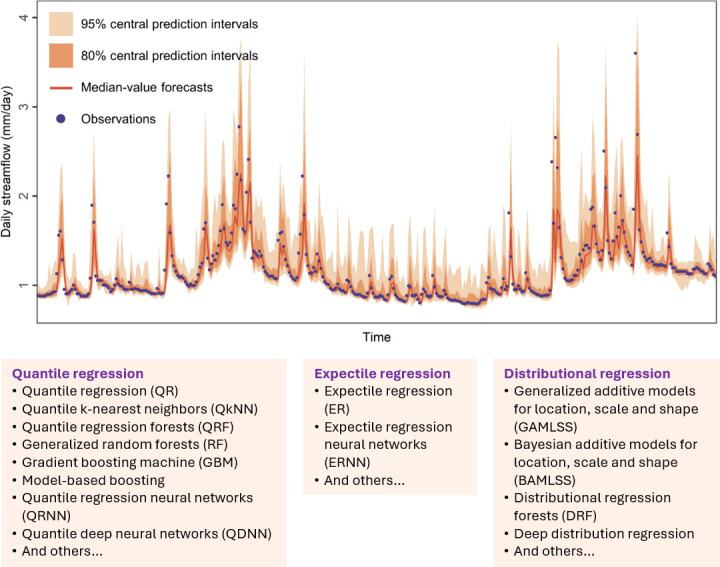
Probabilistic daily one-day ahead machine-learning streamflow forecasts. Median value is indicated in red, 80% (95%) central prediction intervals as dark (light) orange ribbon, and observations as purple circles. Modified from Papacharalampous & Tyralis [35].

Different approaches have been suggested to quantify uncertainty, including the running of Monte Carlo simulations, bootstrapped training samples, Bayesian approximations or modifying the dropout scheme during inference [37]. In neural networks, the dropout technique [89] can be used during testing to generate an ensemble of predictions. By dropping units, ‘thinned’ networks can be trained. Compared with multiparameter ensembles, dropout ensembles are similar to a ‘Bayesian approximation’, offering more reliable but less sharp coverage of prediction intervals, and require only a single calibration run (parameter set), thus limited additional computational cost [37]. Deep ensembles or stochastic variational inference are also increasingly popular in uncertainty quantification. Schreck *et al.* compared evidential neural networks with ensemble techniques to estimate predictive uncertainty. They showed the benefits of the evidential neural networks in terms of their interpretability and computational efficiency [90].

### (d) Uncertainty of XAI methods for model interpretability

The use of post hoc XAI methods on ML models for process understanding can lead to misinterpretations [91,92] partly because there is no clear consensus on what constitutes a valid explanation. As such, many have argued that we should favour models that are interpretable to begin with, rather than post hoc interpretations [91]. However, intrinsically interpretable models sometimes fail to match the predictive performance of more complex ML models, in part because these complex models have access to a larger solution space. For this reason, post hoc XAI methods are often used to give the model posterior interpretability, balancing the need for high performance with the desire for interpretability.

The interpretation of different features in a machine learning model can be highly variable depending on the choice of data sources (and their uncertainties), the type of ML model used, the choices made in the model structure and training and the choice of XAI methods, including specific assumptions and computations inherent in each XAI method [25]. Often, the application of different XAI methods to a single model [93], or even the repeated application of the same XAI method to the same model and input instance [94], can lead to interpretation results that differ to varying degrees. One question is thus whether researchers should combine insights from all interpretation techniques and ‘average’ results to reach a reliable conclusion, or favour specific techniques over others. This domain is highly unregulated, and many researchers use different combinations of techniques, or just one.

**Figure 10.**
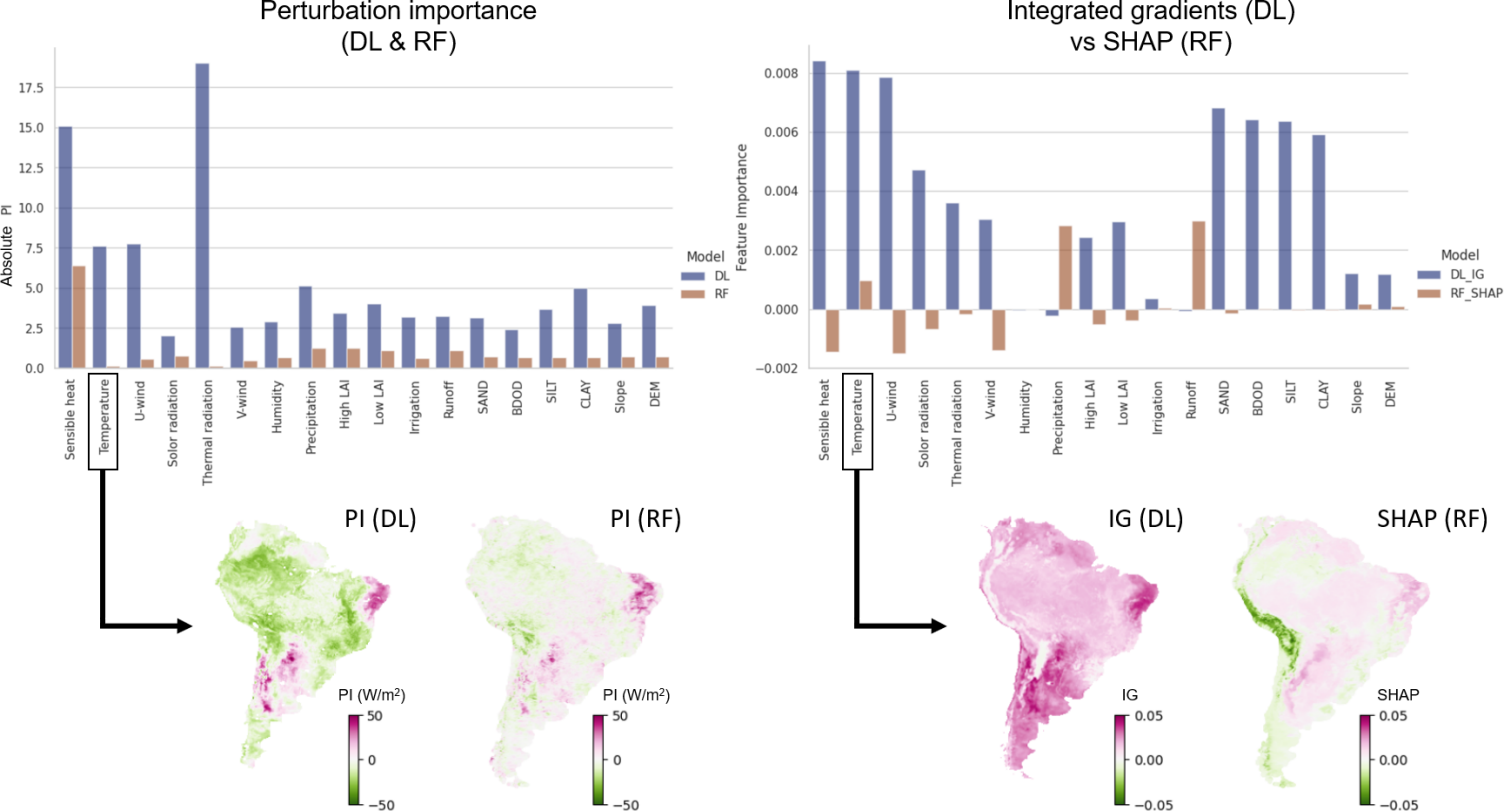
Comparison of instance-level variable importance and attribution in a deep learning (ConvLSTM; DL) and a machine learning (Random Forest; RF) model used to predict land-atmosphere coupling. (Left panels) Perturbation-based importance (PI) of input variables for DL and RF. (Right panels) Feature importance derived from Integrated Gradients (IG) for DL, and SHAP values for RF. The maps indicate spatial attributions for a single variable (temperature). Modified from Huang *et al.* [95].

Huang *et al.* [95], for example, investigated instance-level variable importance in an ML model (Random Forest, RF) and a deep learning model (ConvLSTM), predicting the land atmosphere coupling (LAC) strength, which has been increasing over the last four decades in South America. They compared the outputs of different XAI techniques for both models [95]: perturbation importance (PI) and integrated gradients (IG) for the DL model, and PI and SHAP for the RF model. Although both models exhibited comparable predictive performance, the XAI results were inconsistent (figure 10). The DL model showed higher perturbation importance, suggesting greater sensitivity to perturbation. Additionally, the DL model’s integrated gradients highlighted broader global effects of temperature, while the SHAP values emphasized more localized contributions. Overall, this example highlights the importance of understanding and justifying the choice of specific XAI approaches selected for any given task in hydrology.

Ahmed *et al.* [13] highlight the importance of model interpretability and understanding ML models, so that there is transparency in any conclusions which may impact policy decisions. Moreover, the question of whether XAI results should be interpreted through the lens of pre-existing domain knowledge is a challenge in itself. Promptly disregarding methods where the results are not fully aligned with pre-existing knowledge can limit the potential of hydrological XAI, reinforcing established theories, rather than recognizing the potential of ML as an exciting new approach to reveal novel and possibly counter-intuitive patterns directly from data [25].

## 5. Conclusion: ML and XAI limitations and future recommendations

Common pitfalls of ML in hydrology include issues such as variable selection bias, testing models on the same data they were trained on (resubstitution validation), inconsistent validation across multiple algorithms, and model selection or optimization based on the test set [96]. In the field of large-sample hydrology specifically, based on the current state of the art in ML and XAI, we believe some of the key remaining challenges are as follows.

### (a) Addressing variability in interpretations resulting from different ML models and XAI techniques

Different ML models and XAI approaches can produce conflicting results, and as such can lead to different interpretations [93,95,97]. Rather than considering specific methods to be wrong, and others ‘right’, users need to be aware of the inherent strengths and limitations of each ML model or XAI method. We have attempted to outline some of the strengths and weaknesses of ML/DL models and XAI tools herein. For instance, PDPs can be highly sensitive to variable multicollinearity, and should be assessed alongside potentially more robust approaches such as ALE. Our discussion of these different XAI/interpretability methods aims to provide some guidelines on their respective utility and trustworthiness, a concept which requires co-development strategies with AI users and stakeholders [98]. To increase trust in model results, we recommend that any XAI analysis be repeated by resampling the data, using different subsets of data, varying initial seeds in ML models, or using multiple XAI methods in different runs [99]. This strategy can potentially prevent the finding from being the product of a specific data set, ML model or XAI method, and also allows for estimation of the variability and confidence intervals of the interpretation results.

### (b) Enhancing prediction in data-sparse environments

There has been considerable progress in the field of large-sample hydrology for hydrological prediction in ungauged basins and regions. Many papers have showcased the strengths of ML compared with traditional hydrological models, exploring which model types perform best out of sample, and how different types of out-of-sample prediction (random, geographical, hydrologically separated, temporal or by climate zone) affect generalizability [3]. However, there remain areas for further research, such as: improving the spatial representation of meteorological and geophysical predictors (i.e. moving beyond the use of catchment-averaged values), including spatial dependencies [29], better predicting the most extreme ‘unseen’ events, and evaluating model performance and uncertainty in the most data-sparse regions where many observations remain unavailable or unreliable. Ultimately, increasing data collection in underrepresented regions [100] and better integration of satellite information [88] are both needed to improve global large-sample hydrology.

### (c) Improving predictions in human-modified, bifurcating and nonstationary segments of the global river network

There remains scope to improve our understanding of hydrological drivers in heavily modified or regulated catchments. This includes research on the ability of DL to learn different anthropogenic impacts on hydrological variables, a promising direction given its capacity to infer unmodelled or implicitly represented processes [6]. In regulated basins, LSTMs can outperform process-based hydrological models such as LISFLOOD [101]. Meanwhile, hybrid models that integrate DL with process-based approaches have shown improved performance in simulating reservoir outflows while preserving physical interpretability [102]. The new global river network, GRIT (Global River Topology) [103] also presents an exciting opportunity to generate predictions in data-sparse, human-modified and nonstationary environments. GRIT blends the 30 m Landsat-based river mask from GRWL [104] with elevation-generated streams from the new 30 m FABDEM [105]. It faithfully represents divergent river flows (bifurcations, multi-threaded channels and canals) and can be dynamically updated over time, paving the way to a better representation of global hydrology.

### (d) Developing better multivariate prediction

Predicting multiple dependent variables (outcomes) simultaneously is particularly useful for obtaining more accurate insights in large-sample hydrology. One of the strengths of differentiable modelling is the ability to consider multiple hydrological variables simultaneously. However, an equivalent data-driven approach for handling multivariate hydrological outputs remains an area for further research. Recently, there have been explorations in multi-task learning that incorporate basic hydrological principles, such as water balance, to constrain the relationships between variables computed by ML models [56,57,106]. These studies show promise for multi-variable constraints compared with focusing only on streamflow or a single variable, which may not sufficiently constrain the internal processes in the hydrologic system.

### (e) Rethinking hydrology’s ‘cascade of uncertainty’

The traditional hydroclimatic modelling framework includes multiple sources of uncertainty arising from climate models and other data inputs, through to the hydrological models and post-processing. By contrast, ML models can implicitly handle steps such as downscaling and bias correction of climate forcing data by developing direct relationships between good quality data sources [30,107]. This implicit handling of biases can be seen in cases where higher resolution meteorological inputs (obtained through downscaling and bias correction) do not lead to better hydrological ML model performance, suggesting that the model has already addressed these biases implicitly [29]. We argue that in some cases there is scope for new prediction methods that train ML directly on dynamical forecasts [32] or climate projections [108] to shorten the hydrological modelling chain [32,107]. Despite such methods, however, the uncertainty in streamflow measurements continues to be a fundamental challenge for hydrology [109].

### (f) Improving large-sample methods for hydrological causality

In hydrological research, practitioners—including ourselves—must remain cautious about inferring causality when exploring drivers of hydrological processes. Achieving high predictive accuracy does not necessarily imply valid causal relationships. The persistent challenge of ‘equifinality’, in which different model structures or parameter configurations yield similar results [110], is equally relevant in ML contexts. Recent developments in causal methods for large-sample hydrology have begun to address these issues explicitly [111]. Within this evolving framework, XAI offers hydrologists a powerful tool to interrogate ML models and assess their representation of reality. However, results should always be interpreted with caution, as XAI only reveals the internal mechanics of the ML model, which should not be mistaken for the real world. Although insights derived from XAI should not be mistaken for causal evidence, the correlations and patterns it uncovers can nevertheless provide valuable insights. For example, XAI may reveal that certain predictors previously considered unimportant exert a significant influence on predictions, or that the importance of variables changes unexpectedly in different environmental contexts [25] or over time. Such revelations may prompt hydrologists to reevaluate their assumptions, leading to further testing and targeted studies. Therefore, in most cases where causality is still a challenge, it is preferable to consider XAI findings as hypotheses rather than definitive causal conclusions.

### (g) Working with machine learning experts

While it is valuable for hydrologists to expand their skill set with machine learning techniques, the importance of actively collaborating with machine learning experts cannot be overstated, as is already recognized in other disciplines like ecology [112]. Previous studies have highlighted that successful outcomes often rely on co-authorship between machine learning and hydrological experts [15].

### (h) Learning from our failures

Various challenges arise when implementing ML for large-sample hydrology; these also represent an important step forward. Most papers present only the successes of their ML modelling attempts and the promises of their model architectures, with some briefly mentioned drawbacks in the Discussion sections. However, when it comes to new ML and XAI methods in hydrology, where best-practices are still not well established, it would be highly beneficial to share our ML and XAI difficulties. Sharing our model failures and shortcomings would significantly speed up scientific advancements, both in terms of methods and results. Such efforts are considerably enhanced by making code and data freely available to the research community [113].

Taking these limitations and recommendations into account, we remain highly optimistic on the future of ML and XAI in large-sample hydrology. There is considerable potential to drive future progress in the field, to enhance early warning systems and discover new processes which diverge from our traditional understanding. Here, we are not suggesting a move away from process-based modelling; rather, we highlight the complementary advantages that ML and XAI offer for tackling challenges that traditional methods may struggle with. Looking ahead, we foresee hydrologists widely embracing the nonlinear insights provided by ML, leveraging it as a powerful analytical tool in this data-rich era. We also expect increased cross-disciplinary collaborations, where hydrologists and machine learning experts work together to develop and fine-tune ML and XAI tools specifically designed to meet the diverse needs of different sub-disciplines of hydrology. Overall, we think AI/ML has clear potential for better water resources management in a rapidly changing world.

## Data Availability

This article has no additional data.
